# A framework for rigorous evaluation of human performance in human and machine learning comparison studies

**DOI:** 10.1038/s41598-022-08078-3

**Published:** 2022-03-31

**Authors:** Hannah P. Cowley, Mandy Natter, Karla Gray-Roncal, Rebecca E. Rhodes, Erik C. Johnson, Nathan Drenkow, Timothy M. Shead, Frances S. Chance, Brock Wester, William Gray-Roncal

**Affiliations:** 1 The Johns Hopkins University Applied Physics Laboratory, Research and Exploratory Development Department, Laurel, MD 20723 USA; 2 Sandia National Laboratories, Albuquerque, NM 87185 USA

**Keywords:** Mathematics and computing, Computer science, Human behaviour

## Abstract

Rigorous comparisons of human and machine learning algorithm performance on the same task help to support accurate claims about algorithm success rates and advances understanding of their performance relative to that of human performers. In turn, these comparisons are critical for supporting advances in artificial intelligence. However, the machine learning community has lacked a standardized, consensus framework for performing the evaluations of human performance necessary for comparison. We demonstrate common pitfalls in a designing the human performance evaluation and propose a framework for the evaluation of human performance, illustrating guiding principles for a successful comparison. These principles are first, to design the human evaluation with an understanding of the differences between human and algorithm cognition; second, to match trials between human participants and the algorithm evaluation, and third, to employ best practices for psychology research studies, such as the collection and analysis of supplementary and subjective data and adhering to ethical review protocols. We demonstrate our framework’s utility for designing a study to evaluate human performance on a one-shot learning task. Adoption of this common framework may provide a standard approach to evaluate algorithm performance and aid in the reproducibility of comparisons between human and machine learning algorithm performance.

## Introduction

While progress has been made in artificial intelligence (AI) and machine learning (ML) research towards achieving human performance on perception tasks, one of the biggest challenges facing the field is the creation of algorithms endowed with flexible cognition - the innate ability to balance and switch between multiple tasks that theoretically underpins human general intelligence^1,2,3^. Towards understanding algorithm performance, researchers commonly compare the performance of algorithms under study to that of previously established models (e.g.,^4^) and sometimes to human participants (e.g.,^5,6^). In some cases, when algorithm performance is compared against that of human participants, researchers make exciting claims that the ML algorithm exceeds human performance. While these algorithms undoubtedly advance the field, these comparative results should be approached with caution; when human factors, psychology, or cognitive science research experts, and experts in other fields that study human behavior scrutinize the methods used to evaluate and compare human and algorithm performance, claims that the algorithm outperforms human performance may not be as strong as they originally appeared^7^.

It is not always clear that algorithms fairly outperform human participants on a specified task. These human performance studies may be limited in scope or poorly-controlled (e.g.,^6^). Furthermore, some researchers do not report important information about the human participants used in the study. For example, they may not report how many human participants were tested or the level of expertise that the human performers have in completing the task (e.g.,^8,9^). When human performance is evaluated for use in comparison to algorithms, reporting this information as a key part of the research, as opposed to an afterthought, may aid the research community in assessing not only how accurate algorithms are at the given task, but also what makes human cognition and algorithm intelligence different within the context of the task. This comparison between human and algorithm performance may serve to advance algorithm capabilities. Through these comparisons, researchers may gain inspiration from the ways in which human performers succeed - or fail - at a given task and use this inspiration in the model refinement process. Human-algorithm comparison presents opportunities to generate more accurate claims about the performance of particular algorithms and to gain greater insight into the intricacies of human cognition. In doing so, researchers may find new areas where human cognition fails as well as areas where algorithms must continue to improve.

A framework for evaluating human performance in machine learning is necessary. It is becoming more common for major machine learning conferences to review a reproducibility checklist as part of the submission evaluation process. For example, the 2021 Neural Information Processing Systems (NeurIPS) conference has a section of their reproducibility guidelines dedicated to crowdsourcing and work with human subjects, instituting basic requirements for authors that employ the use of such data including review by relevant ethical authorities when working with human participants and reporting on the task provided to human participants^10–12^. Not all conferences currently require this information about human data to be reported, and a dedicated framework for carrying out human studies for comparison to machine learning performance may aid the machine learning community in the pursuit of producing and reporting these results.

Many instances exist in which human performance is not adequately studied or reported when drawing comparisons to machine learning performance. For instance, the Stanford Artificial Intelligence Index Report^13^ writes that some natural language processing models are able to outperform human ability on language processing tasks^14^, based on the SuperGLUE Benchmark^15^. However, limited information is available about how the human performance baselines were derived. Key pieces of information missing include the number of human participants studied^8,9^, the recruitment method for their research participants^8^, measures of variability between human participants^14–17^, and detailed information about the human task itself (e.g., with a schematic of the task design)^16,17^. Another study referenced in the Report claimed that classifier performance exceeded human performance on an image classification task, however this study used only one human annotator and did not detail the methods used for human evaluation^18^. (To the Report authors’ credit, they do caveat these findings with a statement indicating that these results do not mean that classifier performance exceeds human performance in general).

There are notable instances in which the study of human performance and subsequent comparison with machine learning performance are executed well. In this work, we provide a framework to consolidate the practices found in these instances. Rajalingham et al.^19^ investigated the differences between human and machine learning algorithm behavior on an object recognition task. Examples of practices used in this study that make it an exceptionally good example of human performance evaluation include the use of naturalistic synthetic images due to cited differences in human and algorithm cognition^20^, matched trials and experimental paradigm between human participants and the model evaluation, and following best practices in psychology research studies. These included using stimuli validated by a separate pilot study, recruiting a large subject pool, and controlling for performance strategies such as memorization of task images. Another example of a well-executed study comes from Mohseni et al., who created a benchmark through study of human participants to evaluate model saliency explanations, towards the ultimate goal of providing more interpretable AI^21^. These researchers employed many of the practices we advocate in this work, including a recruiting large human participant pool, completion of relevant ethical reviews, and matching of trials across evaluation groups. These are not the only examples of well-executed and well-reported comparisons between human and algorithm performance; for example, Buetti-Dinh et al. (2009) provide information about the human subject pool under study and justification for the sample subject pool^22^. The specific best practices employed by these well-executed studies and those like them may not be directly applicable for all machine learning researchers and all tasks. As such, we extract higher-level guidelines for completing a rigorous human evaluation that may be applied to a diverse range of machine learning tasks.

In this work, we advocate for the rigorous evaluation of human performance for subsequent comparison to the performance of machine learning models, with the recognition that it is intractable to expect all machine learning researchers to gain the appropriate domain knowledge in psychology to design these comparison studies alone. As such, we outline a set of high-level best practices for the comparison of human performers and machine learning algorithms and provide a demonstration of our approach for human-algorithm evaluation on a one-shot learning task.

The framework has three components. First, the similarities and differences between human and algorithm abilities should be examined with the goal of conceptualizing a test framework that doesn’t unfairly advantage or disadvantage either party (AI/ML or human). Second, specific aspects of the human evaluation should be matched to the algorithm evaluation, including using the same stimuli and trials between evaluation groups. Finally, the evaluation study should be implemented under best practices for psychology research studies, even if the focus of the comparison is not solely in human performance. These practices, such as augmenting experimental data with subjective data and reporting on these results, may aid in validating findings and improving study replicability. This work builds off of that of Firestone 2020, which advocates for three factors when comparing human and machine performance: to “limit machines like humans”, “limit humans like machines”, and “species-species task alignment”^23^. Through our framework, we provide steps to specifically limit humans like machines and align the task of interest between humans and machines, with added information for the machine learning researcher regarding best practices in conducting studies of human performance.

## Guiding principles for successful human evaluation

Comparisons between algorithm and human performance provide a baseline against which algorithm performance may be evaluated. However, it can be challenging to design a human study such that a successful comparison between machine learning and human performance can be made. Towards development of a standardized framework for evaluation, we present three broad guiding principles for designing and implementing a successful evaluation of human performance, towards the goal of aiding the machine learning community in designing high-quality, reproducible studies.

### Design with the differences (and similarities) between human and algorithm cognition in mind

A computer vision algorithm - for example, one trained to recognize pedestrians on the street^24^ - is endowed with the “skills” that it has been trained to develop. In this case, we can count on the algorithm being trained to differentiate human pedestrians from street signs in a crowded scene, but we cannot, for example, expect it to “know” what the street signs surrounding pedestrians say. Additionally, the algorithm doesn’t tire of completing a given task; researchers can ask the algorithm to do many trials multiple times without worrying if performance might degrade due to fatigue.

In contrast to the algorithm, a human driver scanning the road for pedestrians while driving cannot separate what they know about pedestrian appearance and behavior from their knowledge of the meaning of street signs. The human driver uses knowledge that is *outside* the realm of pure visual search to anticipate where pedestrians are likely to appear, increasing their ability to quickly and accurately recognize pedestrians when they see them^25^. Not all of these additional abilities, intertwined with the ability to perform visual search for pedestrians, correlate with positive task performance. For example, the ability to attend to multiple tasks at once may make a driver distracted, leading to a failure in identification of the pedestrian^26^.

In our example above, we illustrate some of the stark cognitive differences between human and machine learning algorithms. The different facets of human cognition are intricately tied together and cannot be easily decoupled even in simple tasks. Algorithms are specially trained for their specific purpose which may aid their performance, but they cannot bring past experience to tasks, which may be at their detriment when compared to human performers. These facts become extremely important when developing a task that accurately compares human and algorithm performance.

When designing a comparison task, it is important to remember these differences in cognitive ability in order to evaluate the two parties fairly. As an example, if a researcher is to evaluate the visual search abilities of humans versus an algorithm, she should bear in mind that human memory is fallible and take action to limit how much she asks her participants to remember throughout the task. Failing to control for this may result in artificially low performance measures for human participants. On the other hand, humans may be advantaged in a particular task if they’re able to bring past experience to the table. This may result in a comparison in which the machine learning performance appears far lower than that of its human counterparts, simply because of inflated human performance.

Attempts to control for the facets of cognition that may be available to humans but not machine learning models may be helpful when attempting to compare human and machine learning performance on a particular specialized task. Some facets of human cognition that a researcher may want to exert a level of experimental control over include language ability, memory (both in terms of past experience and working memory^27^), and attention. The specific cognitive abilities that should be considered when designing the comparison task will depend on the type of algorithm and comparison desired; comparison of human and algorithm performance on speech recognition task may recruit different cognitive functions and have different constraints than a comparison of capabilities on a visual search task, especially depending on the response modality for human participants. Enumeration of the cognitive demands on human participants (e.g., “memory”, “focus”) and the cognitive advantages of human participants (e.g., “reading”, “real-world experience”), even loosely, may aid researchers in designing an evaluation that is balanced to both parties.

### Match trial type and anticipated difficulty between algorithm and human participants

When designing the human evaluation, the stimuli chosen should match those that will be presented to the machine learning algorithm at time of testing. It may not be possible to evaluate both human and algorithm performance on every individual stimulus or trial; however, performance comparison claims will be more robust if main features of the datasets used are matched to be as similar as possible.

Variability in trials can often be helpful to test human and machine performance under a wide variety of conditions. Criteria for trial selection in the final experiment could seek to maximize variability in difficulty level or other features that are expected to influence performance, such as the spatial distribution of target objects in a visual search task. It can also be helpful to screen trials and exclude ones that may be too difficult or frustrating for human participants to complete, as this could lead to data quality issues due to disengagement by human participants.

The evaluation paradigm should also consider known human physical (e.g., visual acuity) and cognitive limitations to ensure that researchers do not unfairly disadvantage human performance on the task, especially due to differences in qualities human participants and machine learning algorithms that are not of research interest. In some cases, this quality of human cognition may be of interest if the goal is to see whether machine learning algorithms can perform better than humans on highly fatiguing tasks. In other cases, however, researchers may be more interested in comparing machine and human performance on one-shot tasks in which measurement of fatigue is not the primary research interest. In this setting, it may be prudent to try to eliminate causes of unnecessary fatigue.

When implementing the study, especially if doing so outside of a research laboratory setting (e.g., on a crowd-sourcing platforms), it is important to consider the ways that human participants will interact with the task. This may influence the trials that are selected for human annotation. For example, on a visual search or identification task, participants may use computers with different screen sizes and browsers with different resolutions. Each of these factors could impact how participants view the stimuli and respond to prompts, especially if the task involves small or blurry images. It is important to ensure that the evaluation paradigm does not put some participants at an unfair disadvantage due to these potential constraints. Collecting data on these constraints such as through surveys asking questions about the equipment participants are using to complete the task and imposing equipment requirements as part of the experiment inclusion criteria can help address these issues, as can ensuring trials are compatible with a wide range of consumer-grade hardware.

### Employ best practices for executing psychology research studies

Our final guiding principle is broader: the machine learning researcher should aim to employ best practices for the execution of psychology and human factors studies when implementing their evaluation of human performance. These best practices include choosing a platform that allows human participants to interact with the task easily, recruiting an appropriate population in terms of population size and demography, conducting internal pilots of the study, collecting supplementary data in addition to main study data, and reporting analysis in terms of the main measure of interest (e.g., accuracy), as well as on other axes (e.g., time to complete the trial, patterns of false positives and false negatives).

Collecting small-scale pilot data may aid researchers in developing their human performance study further by testing that human participants understand what they are being asked to do, the task is not more or less taxing than anticipated, the compensation rate is fair and reasonable, and there are no technical issues that may influence human performance. Reporting measures of variability among human performers, such as amount of agreement, the standard deviation, or a 95% confidence interval on performance is also helpful for interpreting results. Finally, counterbalancing of the trials or, more simplistically, presentation of a random order of trials to each participant, may be helpful for controlling for serial order carryover effects. These practices are common in experimental psychology and may help machine learning experts design a rigorous evaluation of human performance.

Given that many researchers may choose to use a crowd-sourcing platform to conduct their studies, and the movement in the field toward more in-depth reporting of the human experimental design and results when comparing to machine learning algorithms, we provide more in-depth considerations for crowd-sourcing, collection of supplementary data, and respect for human participants below.

#### Considerations for crowd-sourcing

Numerous tools exist for collecting human performance rapidly at scale. Online crowd-sourcing sites such as Amazon Mechanical Turk and Prolific can help researchers collect large numbers of user responses in a short period of time. Crowd-sourcing sites have been commonly used by machine learning communities for generating labeled datasets, and psychologists have long used these sites for performing studies. In the setting of a human performance evaluation, stimulus presentation may be accomplished natively within these platforms and/or externally via providing a link to a custom web application, possibly equipped with a stimulus presentation software (e.g. PsychoPy^28^, PsychToolbox^29^).

When using crowd-sourcing platforms for deployment of comparison studies, it is important to build in methods for monitoring data quality. When participating in an online experiment participants may be more at risk of distraction or lack of attention, which can lead to less reliable data^30^. Fortunately, many research studies have demonstrated that classic psychology experiments can be replicated with high fidelity via online crowd-sourcing methods, especially when methods are built-in for screening out participants with poor data quality^31^. These methods include building attention check trials into the experimental design that can help detect when participants are not paying careful attention to the task and gold standard trials that are easier, or have a clear correct answer, which help assess whether participants answered easy trials correctly^32^.

Using recruitment criteria that favor participants who are likely to give good effort, such as setting a minimum thresholds for number of previous crowd-sourcing tasks completed and percentage of past acceptable data, may aid researchers in finding reliable research participants. Identifying and screening out workers producing poor-quality or inattentive responses is essential to ensuring that the human performance data can be used as a reliable comparison.

#### Collection of demographics and task-related subjective data

Surveying tools such as Qualtrics exist for collecting supplementary data from participants about the difficulty of trials and about the participants themselves, allowing for greater insight into their performance behavior. Collecting demographics and task-related subjective data is not only helpful for determining participant engagement, but also may aid researchers in identifying potential confounds for their results and provide information about the strategies that participants may have used to complete the task. These data are not commonly reported in the machine learning literature, but are helpful for the research community to understand the full picture of who the participants were who completed the task, and what the participants’ experiences were. One established tool for collecting task load data that may be of use to the machine learning researcher is the NASA-TLX^33^.

It is important to consider the human population of interest when designing a successful comparison study, particularly the desired level of relevant domain expertise and experience with the particular task. In some cases, it may be desirable to compare machine learning algorithms to only novices or only experts on a given task, or a combination of the two. Importantly, the participants included in the evaluation should not be members of the research team, as they carry systematic biases about how the task should be executed, which could skew their performance results. Collecting and reporting data about the domain experience level of research participants can bolster the comparison findings.

#### Respect for human participants

Respect for human participants is integral to ethical psychology research and study of human participants for purposes of comparison to algorithm performance is no exception. Researchers can demonstrate their respect for human participants through payment of a fair wage for participants, regardless of whether the study is performed in the laboratory or online, and ensuring that the research protocol is approved by their Institutional Review Board (IRB)^34^ or corresponding regulatory body when necessary. Participants should be informed that they are participating in a research study, be provided an appropriate avenue to contact the research team if they have any problems, and should be informed that they may revoke their consent to participate at any time without penalty.

Additional considerations may be necessary depending on the task demands. Researchers should consider the nature of their stimuli and whether the content is appropriate for all audiences, for example. If the stimuli contain content that some participants may be sensitive to (e.g., violence), appropriate warnings should be provided to participants before they engage with such content. There are many additional ways that researchers can show their human participants respect; researchers may consider contacting a human subjects protection specialist or member of the IRB at their institution for personalized guidance.

## A case study in one-shot learning

We demonstrate our use of the guiding principles outlined in the Guiding on a one-shot learning task. This task was selected to illustrate the utility of our framework because it represents an open problem in machine learning and computer vision research. While is well-established that humans have the ability to learn from limited exemplars^35^, endowing algorithms with this ability remains an open research topic. We present this case study not as a representation of a perfect study, but rather as a stepping stone towards standardization of these comparisons.

**Figure 1:**
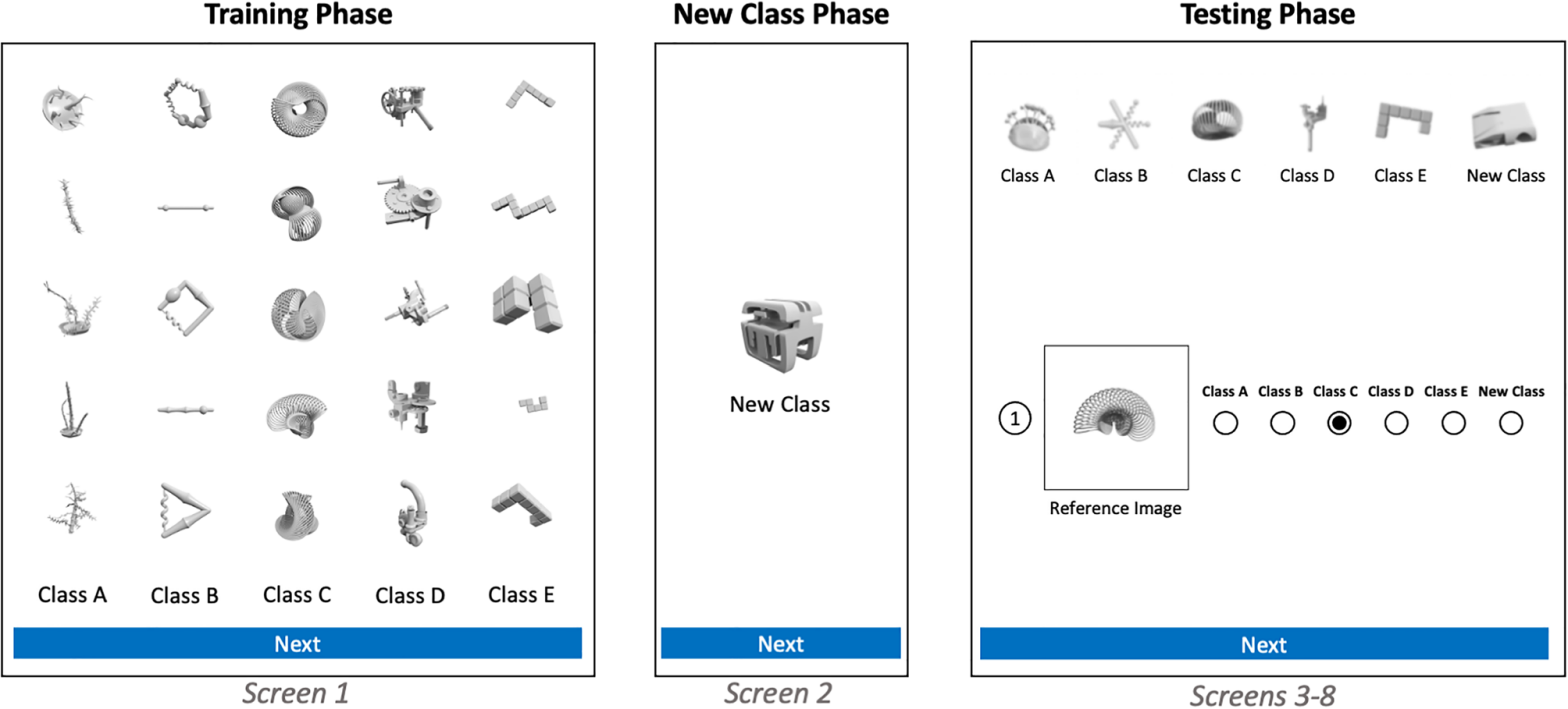
Schematic of our one-shot learning task presented to human participants. At the time of training, participants viewed 5 classes of objects, with 5 examples each. When they progressed to the next screen, they were presented one example of an object from a “new class”. At test time, they viewed 6 consecutive screens with examples from each of the classes, and were asked to categorize to which class they belonged.

A schematic of the task design is shown in Fig. 1, in which the 8 screens comprising one task is shown. Participants first viewed five classes of objects, each with five exemplars. They were then provided one example of an image from a new class of objects. Finally, they were provided six degraded (blurry) example images to remind them of the classes they had previously seen, and asked to which class a new object most likely belonged. Participants had to classify six different objects in total, before a new trial began again with “Screen 1.” A template for Amazon Mechanical Turk of this task can be found in our GitHub repository, as can experimental and supplementary data produced during this experiment.

We demonstrate our use of our framework through controlling for additional aspects of human cognition that were not the objects of study, matching trials between human participants and algorithms and testing a range of difficulty levels with our human participants, and following best practices for executing psychology research studies. These included obtaining approval for the study from our Institutional Review Board (IRB), recruiting a large participant pool, presenting human participants with a set of randomized trials, collecting supplementary research data, and reporting our results with a 95% confidence interval. Through using our framework, we have conducted a study that evaluates human performance for subsequent balanced comparison to the performance of machine learning model trained for the one-shot learning task.

### Consideration of the differences and similarities between human and algorithm cognition

**Figure 2:**
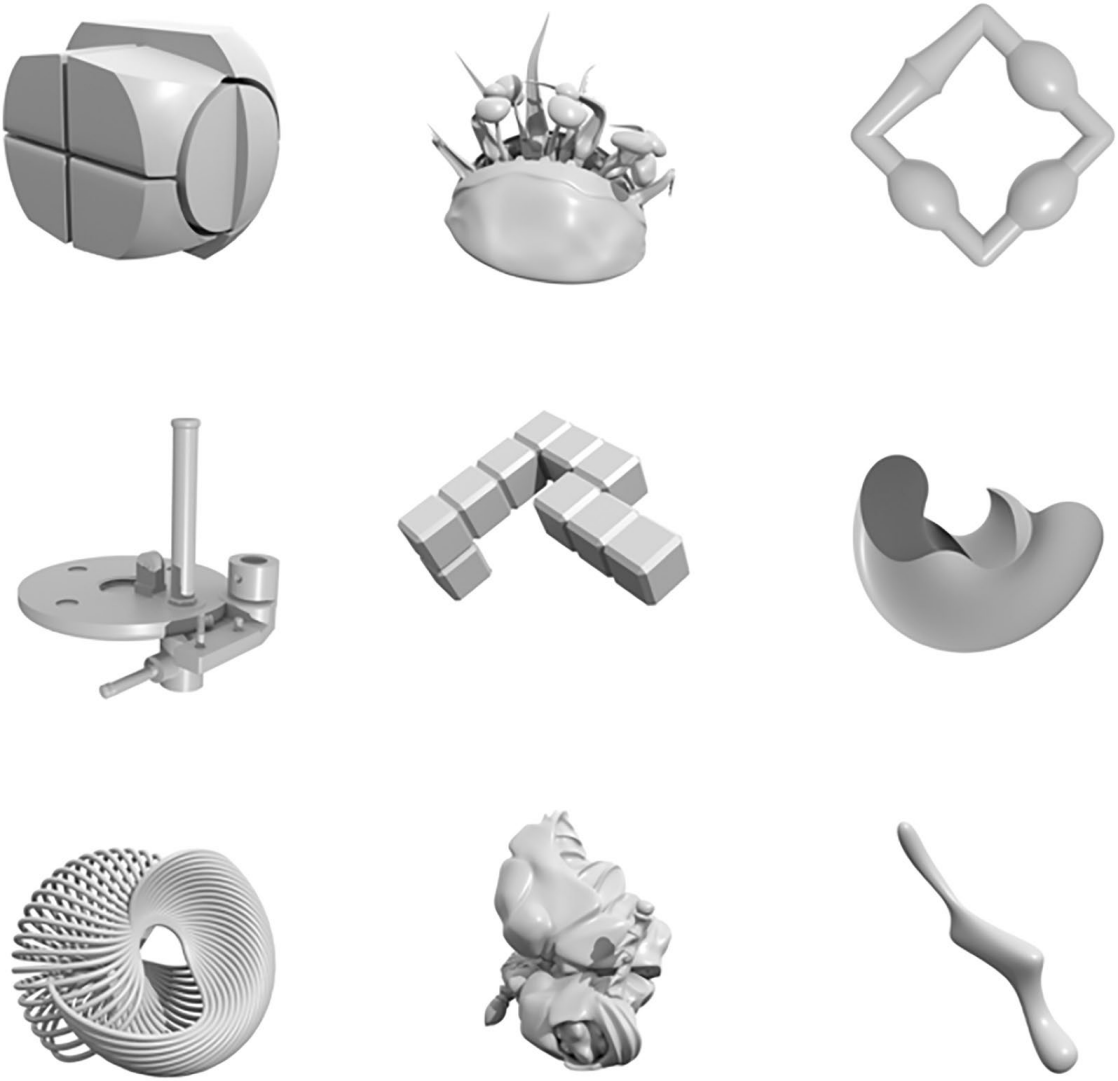
Representative examples of synthetic, computer-generated images used as stimuli in our one-shot learning evaluation. The decision to use synthetic stimuli was made because we aimed to test the human versus algorithm ability to generalize from one experience; as such, we could not use stimuli found in the natural world that participants would have had past experience with.

Of chief importance in this task was controlling for outside experience while testing the ability to generalize from limited exemplars. While the classifier against which we aimed to compare human performance “knows” only the information that it has been trained to know, we cannot exert the same experimental control over our human participants. Therefore, we could not expect to implement a one-shot learning task in human participants using natural images and categories that humans already have experience with, because we would not be able to truly test the human ability to generalize from one instance of a class. Failure to control for outside experience in our human participants would lead to limited internal validity for our findings. To control for this potential confound, we implemented the study with nonsensical, computer-generated objects, such as those in Fig. 2.

It was clear in designing the task that other factors of human cognition were different from algorithm cognition. For instance, while algorithms may be able to encode and store a perfect representation of pixel values in an image, humans have limits to their working memory^36^. Because we were not interested in performance differences on this task that were attributable to differences in working memory capacity, we strove to circumvent this limitation of human cognition by providing degraded “reminder” images to human participants at test time so that they would be able to focus on generalizing from experience, rather than holding information in working memory. Finally, while a computer vision algorithm may have no notion of language, humans are unable to separate their various cognitive capabilities, like their linguistic skills and categorization skills. As such, we refrained from endowing object categories with meaningful names, and instead labeled them with a letter (A, B, C, D, or E) or “New Class” for the purposes of class identification at test time.

Although we took steps to control differences in human and algorithm cognition that we did not want to confound performance, including considering the influence of past exposure, working memory constraints, and the impact of language, perfect control of all of these confounding factors is not possible. This is due to the fundamental challenge that the classifier under study was explicitly designed for a one-shot learning task, in stark contrast to the human mind. While attempts to mitigate differences in human and algorithm cognition contribute to a more fair evaluation of the two, we do not claim that attempts to mitigate these differences result in perfect equivalency between the subject pools. Rather, the ability to draw comparisons is improved. It is possible that our participants directly compared the degraded “reminder” images to the image that they were asked to classify. While we cannot be certain that this strategy was not used, participants were explicitly instructed to study the training images before moving to the testing phase and to only use the blurred reference images as reminders of the classes they had previously seen.

### Matching trials between human participants and algorithms

**Figure 3:**
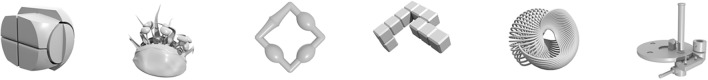
Example of object classes in an easy one-shot learning trial. The differences between classes are more pronounced, allowing for relatively easy discrimination of the defining features of each class.

**Figure 4:**
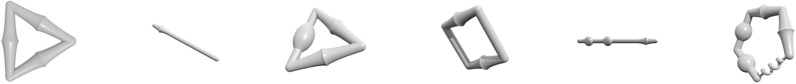
Example of object classes in a hard one-shot learning trial. The differences between classes are less pronounced, demanding greater attention and thought about the defining features of each class.

The synthetic, computer-generated dataset utilized in this comparison allows for the generation of many more unique stimuli than could ever be tested by human participants via varying lighting, shape, orientation, surface texture, and other parameters. Limitations in time, number of participants, and funding were drivers in determining how much data could be collected from human participants, because our goal was to compensate participants with a livable hourly wage for their work and limit the number of trials presented to avoid performance deficits due to fatigue. We provided both the algorithm and human participants trials of two levels of difficulty. A mix of easy (45%) of trials and difficult trials (45%), with the remaining trials (10%) as gold standard and attention check trials were used to ensure that participants were adequately challenged but that they did not become discouraged throughout the course of the experiment. Easy trials were defined as trials that had differences between object classes that were more pronounced. Difficult trials were defined as trials that had differences between classes that were more subtle. Stimuli from an example easy trial can be found in Fig. 3. Note that the differences between the objects in this set of stimuli are quite pronounced, with large variability in overall shape, numbers of components, and surface textures. Stimuli from an example hard trial can be found in Fig. 4. In contrast to the stimuli in the easy trial, the objects in this trial appear to be of the same “family”: they have the same surface texture, and many have the same general shape.

### Implementation of best-practices for executing psychology research studies

In accordance with best practices in psychology studies, we aimed to recruit a large, diverse participant pool via Amazon Mechanical Turk. After performing data quality checks, we included 134 participants in analysis. Although an in-person study would have afforded a higher level of control, this proved infeasible with our current resources. This research was reviewed and approved by the Johns Hopkins Medicine Institutional Review Board (IRB) and all methods were conducted in accordance with the IRB guidelines and regulations for this style of research. Participants were informed in clear language prior to submitting any of their answers that their completion of the task served as informed consent to participate in our research study, per our IRB’s requirements for this type of research. Participants were 18 years of age or older as required by Mechanical Turk and were compensated at a rate of roughly $15 per hour, paid per Mechanical Turk Human Intelligence Task (HIT) completed. Because Mechanical Turk compensates participants per task completed (and not by amount of time spent on a task), the research team set a compensation rate per HIT according to a lower-bound estimate of how many trials a participant could be expected to complete in one hour, as determined by the completion rates for each task by pilot participants. Participants were required to have never participated in one of our Laboratory’s tasks so that they would not have previous experience with our stimuli. 50 responses from different participants were collected per trial to understand how different participants may behave differently and arrive at a generalizable conclusion about human performance on this task.

We identified 200 trials that were representative of the entire synthetic dataset for direct comparison between human participants and machine learning algorithms and the results were extrapolated to the entire dataset. For example, we found that while algorithms show a clear performance decrease when they are tasked with identifying which object belonged in the “new class” (Table 1), human participants showed no such performance decrease (Table 2). This pattern was extrapolated to all trials, given the robustness of the finding. In the comparison shown in Tables 1 and 2, the classifier used was a ResNet18 architecture pre-trained on mini-ImageNet to extract vector embeddings of the input images. For training our one-shot classifier, we embeded each of the exemplars for all training and online classes and saved the corresponding representations. At test time, we embeded our test image and performed k-nearest neighbors search on the saved representations, assigning the label that results from that process to the test image.

**Table 1:** Results from a baseline deep learning algorithm: a ResNet 18 architecture pre-trained on mini-ImageNet. Mean accuracy and 95% confidence intervals are reported across all trial types, calculated using a non-parametric bootstrapping approach. While accuracy on the training classes is comparable to human performance, there is a major drop in performance on the new class. This is due to the algorithm's poor handling of training class imbalance.

Amount of training in target class	Easy trials		Hard trials	
	Mean Accuracy (%)	95% CI	Mean accuracy (%)	95% CI
5 previous examples (training class)	92.7	[90.5, 95.1]	79.6	[76.0, 83.4]
1 previous example (new class)	54.4	[53.4, 55.4]	36.7	[35.7, 37.7]

**Table 2:** Results from human participants. Mean accuracy and 95% confidence intervals are reported across all trial types, calculated using a non-parametric bootstrapping approach. Human performance does not differ with the number of training images to which the participant is exposed. As expected, accuracy on hard trials is lower than accuracy on easy trials.

Amount of training in target class	Easy trials		Hard trials	
	Mean accuracy (%)	95% CI	Mean accuracy (%)	95% CI
5 previous examples (training class)	94.6	[94.3, 94.8]	71.9	[71.4, 72.4]
1 previous example (new class)	95.8	[95.3, 96.3]	70.4	[69.3, 71.6]

**Figure 5:**
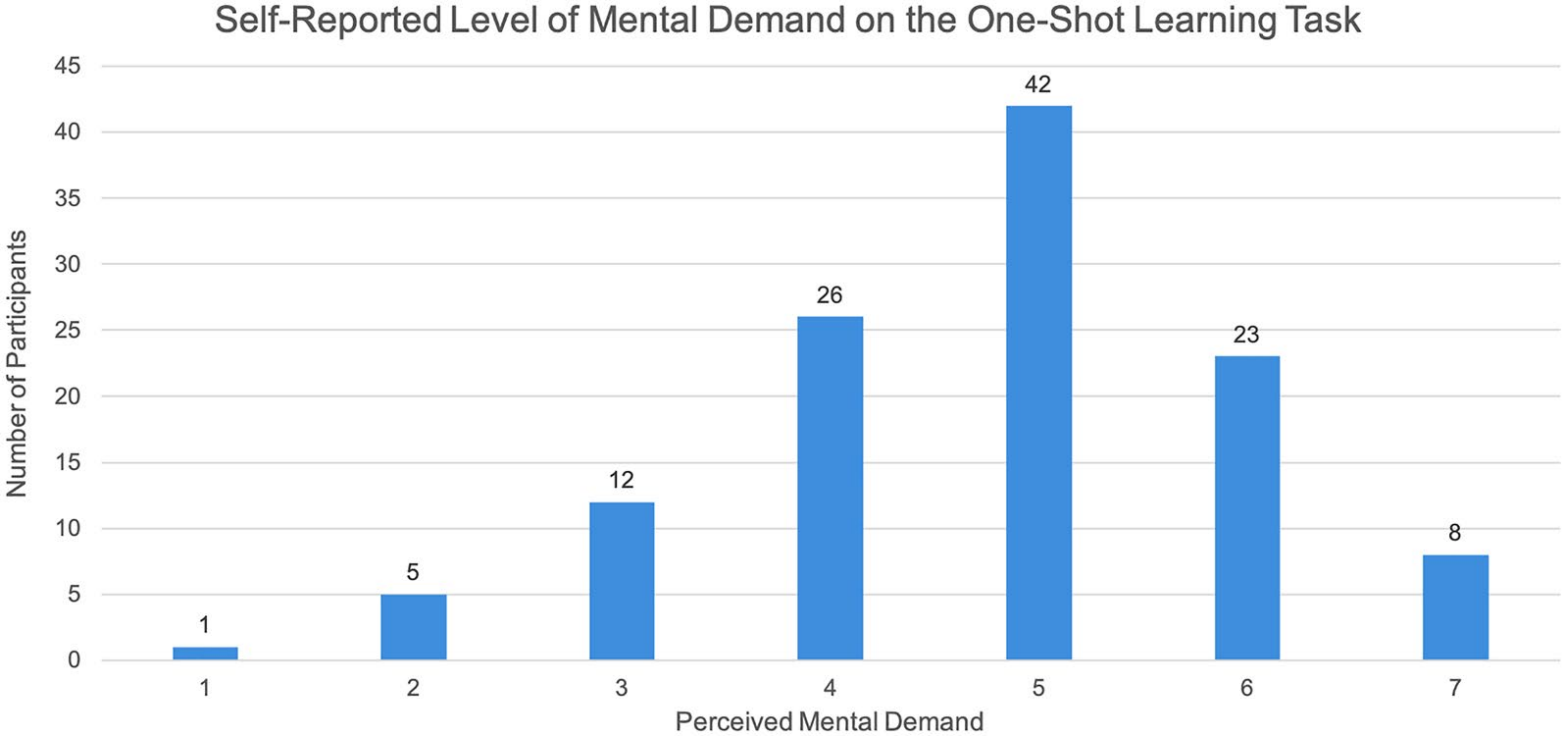
Self-reported level of mental demand required for completing our one-shot learning task (1=Low, 7=High). The distribution of responses indicate that the task was sufficiently difficult to engage participants, but not so difficult that we should be concerned about confounds to human performance.

Finally, although our main outcome of interest was the accuracy with which participants could correctly identify the class from which a particular object was drawn, we collected additional supplementary data common to psychology studies that added context to our findings and, in the event of surprising performance trends, could have helped to identify potential confounds. Our supplementary data included questions relating to the demography of participants, as well as a modified version of the NASA TLX workload questionnaire^33^ to understand how difficult participants found the study and how successful they felt they were at the study, among other measures. The collection of these data helped us to validate that the high performance that we saw in participants is likely not due to the task being too easy, as evidenced by the distribution of self-reported degree of mental demand (Fig. 5).

## Discussion

In this work, we presented 3 guiding principles for standardizing the comparison between human and algorithm performance on common machine learning tasks. First, care must be taken in designing the comparison to consider the  strengths and weaknesses of human participants and the algorithm, to ensure that the evaluation paradigm is as fair as possible. Second, the human and algorithm evaluation should be matched as closely as possible, including matching the specific trials that human performers and the algorithms complete, such that a direct comparison of human and algorithm performance can be made. Third, the comparison must be meticulously implemented, including the collection of an adequate sample of task-naíve human participants, augmenting experimental data with subjective data such as ratings of task difficulty, and the execution of statistically rigorous comparisons. Failure to consider these elements can result in an unfair comparison between machine learning algorithms and human capabilities and could lead researchers to artificially under- or overestimate algorithm performance. We showed our implementation of these principles in our comparison of human and machine performance on a one-shot learning task.

This framework is limited in its ability to directly prescribe solutions for every individual type of machine learning-to-human comparison that a researcher may want to implement. Furthermore, researchers may be limited in their ability to implement the recommendations set forth herein, given funding, participant, and laboratory constraints. With the acknowledgement that adopting a common framework will require community participation and group iteration, this work is presented as a first step towards a common, standardized framework. This framework should be updated with input from the community to ultimately include best practices from a variety of laboratories, research settings, and analysis aims for the particular task setting.

A framework for measuring human performance is necessary for completing comparative evaluations of human and machine learning, but not sufficient alone to ensure a fair comparison study. In this work, we have focused on reducing bias in favor of or against human performance. It also important to consider the opposite potential bias: for or against machine learning algorithm performance, such as the types of biases discussed in^37^. Furthermore, *performance* may not always be the right criterion to compare against. Performance refers to the ways in which the parties under comparison express their knowledge, which is notably different from a framework to assess or compare competence: the knowledge and ability that agents have. This distinction between performance and competence is important for drawing comparisons between the “species” of machine learning and humans that go beyond strict demonstration of performance, which may be the goal of some comparison studies^23^.

Rigorous evaluation of human performance on tasks used to evaluate machine learning algorithms will help researchers to understand not only how well humans perform on these tasks, but also the methods that humans use, the ways in which humans fail, and the ways in which they succeed. Knowledge of how humans perform on these tasks may provide machine learning and artificial intelligence researchers the data they need to make decisions on what it means to create biologically-inspired machine learning algorithms: whether the system must achieve human performance to be considered intelligent, and whether the machine must approach the problem in a similar way to the human, demonstrating similar success and error patterns. A unified framework for performing human evaluation will aid machine learning researchers in designing their studies of human performance and reporting their results, which may aid them in reporting accurate and replicable comparisons to human performance and provide practical tools for meeting the demands that major machine learning conferences are now instituting regarding reporting on human performance.

## Data Availability

The one-shot learning task template for use on Amazon Mechanical Turk, experimental data, and supplementary data for the case study implemented in this manuscript can be found at our GitHub: https://github.com/aplbrain/human-performance-evaluation.

## References

[1] Humphreys LG (1979). The construct of general intelligence. Intelligence.

[2] Barbey AK (2018). Network neuroscience theory of human intelligence. Trends Cognit. Sci..

[3] Pennachin C, Goertzel B (2007). Contemporary Approaches to Artificial General Intelligence.

[4] Ding, D., Hill, F., Santoro, A. & Botvinick, M. Object-based attention for spatio-temporal reasoning: Outperforming neuro-symbolic models with flexible distributed architectures. arXiv: 2012.08508, (2020).

[5] Russakovsky O (2015). ImageNet large scale visual recognition challenge. Int. J. Comput. Vis..

[6] He, K., Zhang, X., Ren, S. & Sun, J. Delving deep into rectifiers: Surpassing human-level performance on imagenet classification. In *Proceedings of the 2015 IEEE International Conference on Computer Vision (ICCV)*, ICCV ’15, 1026–1034, 10.1109/ICCV.2015.123 (IEEE Computer Society, 2015).

[7] Strickland E (2019). Ibm watson, heal thyself: How ibm overpromised and underdelivered on ai health care. IEEE Spectr..

[8] Pilehvar, M. T. & Camacho-Collados, J. Wic: 10, 000 example pairs for evaluating context-sensitive representations. *CoRR* arXiv:abs/1808.09121 (2018).

[9] Zhang, S. *et al.* Record: Bridging the gap between human and machine commonsense reading comprehension. *CoRR* arXiv:abs/1810.12885 (2018).

[10] Pineau J (2021). Improving reproducibility in machine learning research. J. Mach. Learn. Res..

[11] Foundation, N. I. P. S. Neurips 2021 paper checklist guidelines.

[12] Foundation, N. I. P. S. Ethics guidelines.

[13] Zhang, D. *et al.**The Artificial Intelligence Index Report 2021* (Stanford Institute for Human-Centered Artificial Intelligence, 2021).

[14] He, P., Liu, X., Gao, J. & Chen, W. Deberta: Decoding-enhanced BERT with disentangled attention. *CoRR* arXiv: abs/2006.03654, (2020).

[15] Wang, A. *et al.* Superglue: A stickier benchmark for general-purpose language understanding systems. *CoRR* arXiv:abs/1905.00537, (2019).

[16] Nangia, N. & Bowman, S. R. Human vs. muppet: A conservative estimate of human performance on the GLUE benchmark. *CoRR* arXiv:abs/1905.10425 (2019).

[17] Khashabi, D., Chaturvedi, S., Roth, M., Upadhyay, S. & Roth, D. Looking beyond the surface: A challenge set for reading comprehension over multiple sentences. In *Proceedings of the 2018 Conference of the North American Chapter of the Association for Computational Linguistics: Human Language Technologies, Volume 1 (Long Papers)*, 252–262, 10.18653/v1/N18-1023 (Association for Computational Linguistics, 2018).

[18] He, K., Zhang, X., Ren, S. & Sun, J. Delving deep into rectifiers: Surpassing human-level performance on imagenet classification. In *2015 IEEE International Conference on Computer Vision (ICCV)*10.1109/iccv.2015.123 (2015).

[19] Rajalingham, R. *et al.* Large-scale, high-resolution comparison of the core visual object recognition behavior of humans, monkeys, and state-of-the-art deep artificial neural networks. *J. Neurosci.* **38**, 7255–7269, 10.1523/JNEUROSCI.0388-18.2018 (2018). https://www.jneurosci.org/content/38/33/7255.full.pdf. 10.1523/JNEUROSCI.0388-18.2018 PMC6096043 30006365

[20] Pinto, N., Cox, D. D. & Dicarlo, J. J. Why is real-world visual object recognition hard? *PLoS Comput. Biol.* **4**, 10.1371/journal.pcbi.0040027 (2008). 10.1371/journal.pcbi.0040027 PMC2211529 18225950

[21] Mohseni, S., Block, J. E. & Ragan, E. Quantitative evaluation of machine learning explanations: A human-grounded benchmark. In *26th International Conference on Intelligent User Interfaces*10.1145/3397481.3450689 (2021).

[22] Buetti-Dinh A (2019). Deep neural networks outperform human expert’s capacity in characterizing bioleaching bacterial biofilm composition. Biotechnol. Rep..

[23] Firestone, C. Performance vs. competence in human–machine comparisons. In *Proceedings of the National Academy of Sciences* **117**, 26562–26571, 10.1073/pnas.1905334117 (2020). https://www.pnas.org/content/117/43/26562.full.pdf. 10.1073/pnas.1905334117 PMC7604508 33051296

[24] Agrawal, P. & Brahma, P. P. Single shot multitask pedestrian detection and behavior prediction. *arXiv* (2021). arXiv:2101.02232.

[25] Kristjánsson Á (2016). Priming of visual search facilitates attention shifts: Evidence from object-substitution masking. Perception.

[26] Savage SW, Potter DD, Tatler BW (2020). The effects of cognitive distraction on behavioural, oculomotor and electrophysiological metrics during a driving hazard perception task. Accident Analysis & Prevention.

[27] Baddeley A (1992). Working memory. Science.

[28] Peirce J (2019). PsychoPy2: Experiments in behavior made easy. Behav. Res. Methods.

[29] Kleiner M (2007). What’s new in psychtoolbox-3. Perception.

[30] Buhrmester, M., Kwang, T. & Gosling, S. D. Amazon’s mechanical turk: A new source of inexpensive, yet high-quality, data? *Perspect. Psychol. Sci.* **6**, 3–5, 10.1177/1745691610393980 (2011). PMID: 26162106, 10.1177/1745691610393980 26162106

[31] Buchanan EM, Scofield JE (2018). Methods to detect low quality data and its implication for psychological research. Behav. Res. Methods.

[32] Paolacci, G., Chandler, J. & Ipeirotis, P. G. Running experiments on mechanical turk. *Judgm. Decis. Making* **5** (2010).

[33] Hart, S. G. & Staveland, L. E. Development of nasa-tlx (task load index): Results of empirical and theoretical research. In Hancock, P. A. & Meshkati, N. (eds.) *Human Mental Workload*, vol. 52 of *Advances in Psychology*, 139 – 183, 10.1016/S0166-4115(08)62386-9 (North-Holland, 1988).

[34] Graber, M. A. & Graber, A. Internet-based crowdsourcing and research ethics: the case for irb review. *J. Med. Ethics* **39**, 115–118, 10.1136/medethics-2012-100798 (2013). https://jme.bmj.com/content/39/2/115.full.pdf. 10.1136/medethics-2012-100798 23204319

[35] Weaver J (2015). How one-shot learning unfolds in the brain. PLoS Biol..

[36] Cowan, N. *Working Memory Capacity: Classic Edition*. (Taylor & Francis, 2016).

[37] Buckner, C. J. Black boxes, or unflattering mirrors? Comparative bias in the science of machine behavior. *Br. J. Philos. Sci.*10.1086/714960 (2021).

