# Author Correction: A framework for rigorous evaluation of human performance in human and machine learning comparison studies

**DOI:** 10.1038/s41598-022-15857-5

**Published:** 2022-07-07

**Authors:** Hannah P. Cowley, Mandy Natter, Karla Gray-Roncal, Rebecca E. Rhodes, Erik C. Johnson, Nathan Drenkow, Timothy M. Shead, Frances S. Chance, Brock Wester, William Gray-Roncal

**Affiliations:** 1 The Johns Hopkins University Applied Physics Laboratory, Research and Exploratory Development Department, Laurel, MD 20723 USA; 2 Sandia National Laboratories, Albuquerque, NM 87185 USA

Correction to: *Scientific Reports* https://doi.org/10.1038/s41598-022-08078-3, published online 31 March 2022

The original version of this Article contained errors, where Reference 7 was incorrectly cited and omitted. As a result, in the Introduction,

“In some cases, when algorithm performance is compared against that of human participants, researchers make exciting claims that the ML algorithm exceeds human performance (e.g.,^7^).”

now reads:

“In some cases, when algorithm performance is compared against that of human participants, researchers make exciting claims that the ML algorithm exceeds human performance.”

“For example, they may not report how many human participants were tested or the level of expertise that the human performers have in completing the task (e.g.,^7^).”

now reads:

“For example, they may not report how many human participants were tested or the level of expertise that the human performers have in completing the task (e.g.,^8, 9^).”

“These researchers employed many of the practices we advocate for in this work, including a recruiting large human participant pool, completion of relevant ethical reviews, and matching of trials across evaluation groups.”

now reads:

“These researchers employed many of the practices we advocate in this work, including a recruiting large human participant pool, completion of relevant ethical reviews, and matching of trials across evaluation groups. These are not the only examples of well-executed and well-reported comparisons between human and algorithm performance; for example, Buetti-Dinh et al. (2009) provide information about the human subject pool under study and justification for the sample subject pool^22^.”

The original Article has been corrected.

